# Abatacept as a Therapeutic Option for Rheumatoid Vasculitis

**DOI:** 10.7759/cureus.2506

**Published:** 2018-04-19

**Authors:** Layth Al Attar, Timothy Shaver

**Affiliations:** 1 Internal Medicine, University of Kansas School of Medicine - Wichita, Wichita, USA; 2 Rheumatology, University of Kansas School of Medicine - Wichita, Wichita, USA

**Keywords:** rheumatoid arthritis, vasculitis, immunotherapy

## Abstract

Abatacept is a fusion protein composed of the fragment crystallizable region (Fc region) of the immunoglobulin IgG1 fused to the extracellular domain of cytotoxic T-lymphocyte-associated protein 4. Our patient presented with lower extremity purpura in the setting of rheumatoid arthritis and common variable immunodeficiency disease. A biopsy of cutaneous lesions confirmed the etiology of rheumatoid vasculitis. Although rituximab is the recommended treatment, it has the potential to exacerbate immunodeficiency. The cutaneous lesions responded well to abatacept after failure to respond to other treatment modalities. This case is the first, to our knowledge, to be reported in North America. Our case may encourage extensive clinical trials on abatacept as a treatment option.

## Introduction

Rheumatoid arthritis (RA) is a systemic inflammatory disorder of unknown etiology primarily affecting the synovium. Extra-articular manifestations occur in about 40 percent of patients with RA [1]. Cutaneous vasculitis, a clinical manifestation of rheumatoid vasculitis (RV), develops in 5.4 percent of patients with RA [2]. Rituximab has been shown to be effective in inducing remission of RV [3]. It is also documented to be effective in treatment of cutaneous manifestations [4]. On the contrary, abatacept use has been associated with RV. It has been reported that RV may present as a new onset while on abatacept, resolving after being treated with Rituximab [5]. The case we are presenting is of RV managed with abatacept that led to resolution of symptoms.

## Case presentation

We present a case of a 68-year-old Caucasian female known to have multiple comorbidities: seropositive rheumatoid arthritis, common variable immunodeficiency, hypertension, hypothyroidism, and osteoporosis. The patient was diagnosed with rheumatoid arthritis in 2013. She developed cutaneous vasculitis, confirmed by biopsy performed by her primary care physician in May, 2015. She was initially treated with mycophenolate, hydroxychloroquine, and prednisone with initial improvement. The prednisone was tapered off, but she then developed worsening of joint symptoms and synovitis. For this reason, she was switched to methotrexate. Six months later, leflunomide was substituted due to hair loss and inadequate disease control on methotrexate. It was several months later that her cutaneous lesions worsened again. The decision was made to start the patient on abatacept. Arrangements were made with required laboratory testing and screening prior to starting abatacept. On the following visit, the patient was given abatacept 10 mg/kg intravenous infusion with concurrent therapy of leflunomide, hydroxychloroquine, and prednisone. The patient was reevaluated after two weeks of infusion, with clearing of lesions several days after the first dose of abatacept. On her four-month follow-up visit, the patient continued to demonstrate a satisfactory response to this therapy. Prednisone was discontinued, and the patient was kept on abatacept, hydroxychloroquine, and leflunomide. The patient returned to office a month later without any recurrence of her cutaneous lesions.

## Discussion

This was a case of cutaneous vasculitis diagnosed based on biopsy findings. Cutaneous vasculitis is an extra-articular manifestation associated with significant complications. These complications may range from palpable purpura, ulcers, to ischemic lesions [6-7]. The severity of complications depends on the size of vessels involved. The complexity of our case stems from refractoriness to standard therapy, prompting investigation into other treatment options. This prompt search of other treatment option. In our literature review, we found one case of RV responding to abatacept [8]. Consideration of presented comorbidities added limitations to treatment options. Abatacept achieves its effect by binding to the costimulatory molecules CD80 and CD86 on antigen-presenting cells (APC), thereby blocking interaction with CD28 on T-cells [9]. Utilizing abatacept’s therapeutic mechanism was shown to be beneficial in treatment of our patient’s cutaneous vasculitis.

Cutaneous vasculitis presents as lower extremity purpura. It is managed with glucocorticoids and/or immunosuppressive agents such as cyclophosphamide depending on presentation severity. The recent development of biologic response modifiers showed potential to improve outcomes. Rituximab is considered the current biologic agent of choice for rheumatoid vasculitis treatment [3]. Rituximab binds CD20 on B-lymphocytes and induces B-cell death via natural killer cells, often resulting in hypogammaglobulinemia. This would increase the potential to worsen common variable immunodeficiency. Levy R et al. [10] recommends monitoring serum immunoglobulin levels after initiation of rituximab. Abatacept inhibits T-cell activation by binding to CD80 and CD86 on antigen-presenting cells to interfere with signaling via CD28 [9]. This alternative mechanism of action would preserve B-cell function in our patient.

## Conclusions

In conclusion, complex pathophysiology underlies many rheumatologic disorders, providing us an opportunity to explore new treatment options. Newly developed biologic response modifier therapies open up a wider array of possibilities in this regard. While it is premature to recommend the routine use of abatacept in this clinical setting, our case could serve as an impetus for more extensive clinical trials of this agent in the treatment of rheumatoid vasculitis.
